# ASCO update: lung cancer

**DOI:** 10.1007/s12254-017-0373-x

**Published:** 2017-12-01

**Authors:** Gudrun Absenger, Jasmin Terzic, Angelika Bezan

**Affiliations:** Klinische Abteilung für Onkologie, LKH-Univ. Klinikum Graz, Auenbruggerplatz 15, 8036 Graz, Austria

**Keywords:** Carcinoma, non-small-cell lung, Carcinoma, small-cell lung, Lung neoplasms, Immunotherapy, Treatment algorithms

## Abstract

In the past few years there have been major changes in the treatment landscape in oncology; lung cancer is affected by those changes like almost no other solid tumor. The rise of further second- and third-line tyrosine kinase inhibitors offers sequential therapy for patients with mutated non-small-cell lung cancer. Immunotherapy has found its way into clinical routine and presents us with new challenges in managing side effects, evaluating treatment response and deciding on how long we treat our patients. The treatment algorithm of lung cancer has changed in the last month and further practice-changing trials are coming up, so treating lung cancer patients shows nowadays a more challenging perspective with the possibility of subsequently applied individual therapies. This article provides a brief overview of the highlights presented at the ASCO (American Society of Clinical Oncology) annual meeting this year in Chicago.

## Non-small-cell lung cancer (NSCLC)

### EGFR-mutated NSCLC

Approximately 11% of Caucasian patients with NSCLC harbor activating EGFR (epidermal growth factor receptor) mutations and first-line treatment with EGFR-targeted tyrosine kinase inhibitors (TKI) have been proven to be superior in comparison to chemotherapy in patients with metastatic disease [1–3]. In the adjuvant setting, the current standard of care is adjuvant chemotherapy. The Chinese CTONG trial compared adjuvant TKI therapy with gefitinib for two years to the standard of care with 4 cycles of cisplatin/vinorelbine in patients with EGFR-mutated lung cancer. The median disease-free survival was statistically significant better in the gefitinib arm (28.7 months vs 18 months, HR 0.60, *p* = 0.005) and thereby the study met its primary endpoint. However, when adjuvant treatment with gefitinib was stopped after 24 months, the Kaplan–Meier curves converged again so gefitinib maybe just delays recurrence instead of leading to higher cure rates. In all, 65% of patients had N2 disease; in the smaller proportion of patients with N1 disease there was no statistically significant difference between the two treatment arms in subgroup analysis. Further follow-up needs to be awaited for overall survival analysis. Up to now, these data are too immature to change the standard of care.

The phase III ARCHER trial randomized patients with EGFR-mutated lung cancer to first-line treatment with either dacomitinib, a second generation EGFR-targeted TKI or gefitinib as the standard of care. With a longer median progression-free survival (PFS) of 14.7 months in the dacomitinib arm versus 9.2 months in the gefitinib arm the primary endpoint was met (HR 0.59, *p* < 0.0001). However, in this trial patients with brain metastases were excluded which seems not practicable because the central nervous system (CNS) is a common site for metastases in EGFR-mutated patients. Furthermore, the incidence of severe adverse events was more frequent in the dacomitinib arm (acne and diarrhea), requiring dose reduction in 66.1% of patients vs 8% in the control arm. In addition, the study included mainly Asian patients (74.9%) and in the subgroup analysis of non-Asian patients there was no significant difference in PFS.

Osimertinib, a third generation TKI is approved for treatment of patients with advanced EGFR T790M-mutant NSCLC who had progressive disease after EGFR-targeted TKI therapy. In a prespecified subgroup analysis of the AURA 3 trial in patients with brain metastases, osimertinib showed an CNS overall response rate (ORR) of 70% compared to 31% with platinum-based doublet chemotherapy (OR 5.13, *p* = 0.015). The median PFS in the CNS was significantly longer with osimertinib than with chemotherapy (11.7 months vs 5.6 months; HR 0.32, *p* = 0.004). These results underline the value of osimertinib as second-line treatment in EGFR T790M mutated patients. In addition, the FLAURA trial, presented at this year’s EMSO meeting, compares osimertinib with two first generation TKIs (gefitinib or erlotinib) in treatment naïve patients with EGFR exon 19 or 21 mutations. The primary endpoint of the study was met; the median progression-free-survival was 18.9 months compared to 10.2 months (HR 0.46, *p* < 0.0001). The benefit in progression-free survival was consistent across all subgroups, including patients with and without brain metastases.

### ALK-mutated NSCLC

**Fig. 1:**
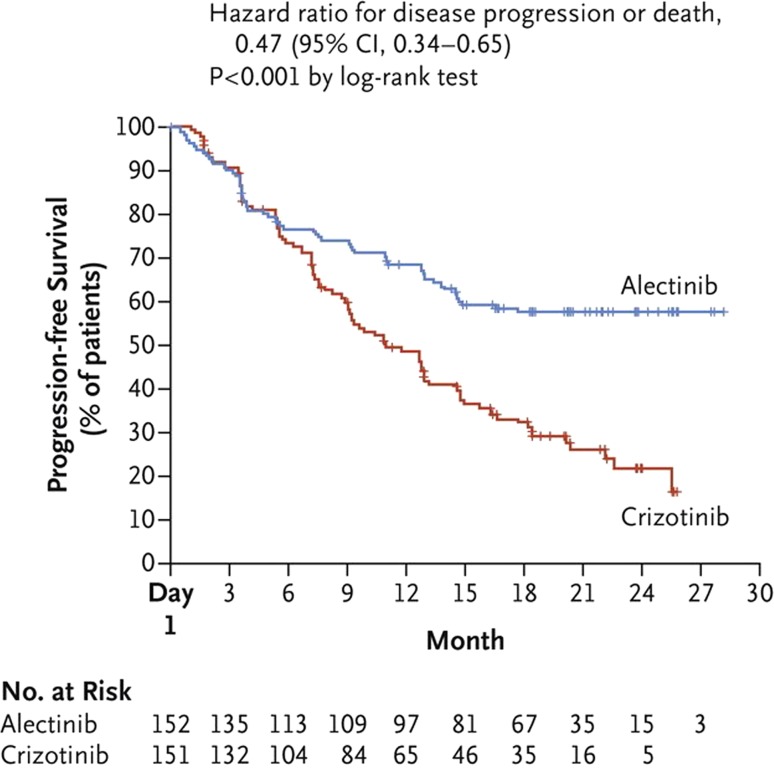
Progression-free survival primary endpoint (ITT Population) [5]

NSCLC with EML4-ALK translocation (echinoderm microtubule associated protein-like4 anaplastic lymphoma kinase) can be found in around 5% of lung cancer patients and is characterized by a high risk of developing brain metastases. In the phase III ALEX trial, treatment naïve patients with stage IIIB or IV NSCLC with ALK rearrangement were randomly assigned to receive alectinib, a second generation ALK inhibitor or crizotinib, the current standard of care. Alectinib extended the median time to progression by about 15 months (median PFS 25.7 vs 10.4 months) and thereby reduced the risk of cancer progression by 53% (HR 0.47, *p* < 0.0001) (Fig. 1). Overall survival analysis is currently considered immature. While both treatments cross the blood–brain barrier, alectinib was more effective in preventing brain metastases. At 12 months, the incidence of brain metastases was much lower with alectinib than with crizotinib (9% vs 41%, HR 0.16, *p* < 0.0001). These results go along with the J‑ALEX trial involving Japanese treatment naïve patients with ALK-positive disease [4]. In addition, alectinib showed a more favorable safety profile. Taken together alectinib seems to be the new standard of care for first-line treatment of patients with ALK-positive NSCLC [5].

## Immune checkpoint inhibition

The ASCO (American Society of Clinical Oncology) 2017 was not the meeting of large Phase III trials in immunotherapy. Beside updates of the practice changing trials like the Keynote 024, managing therapies with immune checkpoint inhibitors was an important topic. Trials discuss questions like treatment duration and dealing with patients who developed immune-related adverse effects (irAE).

## Updated results: Keynote 024 and Keynote 021

Julie R. Brahmer reported updated OS and PFS2 of the Keynote 024 trial based on 19-month median follow-up. The phase III trial compared pembrolizumab with the investigator’s choice of chemotherapy as first-line treatment for patients with advanced NSCLC and PD-L1-tumor proportion score (TPS) ≥ 50% [6]. Preliminary data presented at ESMO last year showed a significant improvement in PFS for pembrolizumab. PFS2 is defined as “time from randomization to objective tumor progression on next-line treatment or death from any cause.” Median PFS2 was substantially improved for pembrolizumab (18.3. vs. 8.6 months, HR 0.54). Survival showed an 18-month OS rate of 61.2% in the pembrolizumab group compared to 43.0% in the chemotherapy group. This data support the use of pembrolizumab for first-line treatment in patients with NSCLC PD-L1-TPS ≥ 50%.

The randomized phase II Keynote 021 trial showed that adding pembrolizumab as third agent to carboplatin/pemetrexed in the first-line advanced NSCLC setting significantly improve ORR (55% vs 29%, *p* = 0.0016) and PFS (HR 0.53, *p* = 0.01) [7]. The toxicity profile, especially grade 3–4 AEs, was manageable (39% vs. 26%). Notablely, PD-L1 negative patients showed a response of approximately 60%. This highlights that the combination of a checkpoint inhibitor and chemotherapy is safe and promising and led to FDA approval of the combination of pembrolizumab and chemotherapy as first-line treatment.

## Treatment beyond progression and managing side effects

The phase III OAK trial enrolled 1225 patients with previously treated NSCLC and randomized them to intravenous atezolizumab (1200 mg every 3 weeks) or docetaxel (75 mg/m^2^ every 3 weeks; [8]). The data were already presented at the last ESMO meeting at Copenhagen with a meaningful survival benefit of atezolizumab over chemotherapy. This year’s ASCO meeting addressed the question of atezolizumab treatment beyond progression (TBP) defined by post progressive disease (PD) tumor regression, OS and safety. Post-PD-regression can result from response due to tumor immune infiltration or delayed response, reducing reliability of RECIST 1.1 as an indicator of treatment failure. Atezolizumab beyond progression showed subsequent response in 7% of patients and stable disease in 49% of patients. The study showed that continuing atezolizumab beyond PD was associated with a prolonged clinical benefit, 12.7 months OS compared with 8.8 months OS for those patients treated with other anticancer treatments post PD. These data support the treatment strategy of continuing atezolizumab beyond PD until loss of clinical benefit in patients, regardless of the level of PD-L1 expression.

In melanoma, a correlation between development of irSE (immune-related side effects) and clinical benefit has been suggested. Owen et al. assessed this question for NSCLC patients treated with nivolumab in a retrospective review and found that patients with irSE had longer median OS (13.2 vs 5.8 months, *p* = 0.018; [9]). Another trial evaluated restart of immunotherapy after Grade 2 or higher irAE. In patients who develop irAEs, re-treatment with anti-PD (L)-1 therapy was associated with recurrent or new irAEs in half of the patients, and was more common in early onset irAEs [10].

## Small cell lung cancer (SCLC)

The CheckMate 032 phase I/II study showed that the treatment with nivolumab alone or in combination with ipilimumab resulted in durable responses in patients with previously treated SCLC. In the non-randomized cohort of this trial, the median OS was 4.1 months for patients receiving monotherapy and 7.8 months for the combination arm, resulting in a higher incidence of adverse events. At 2 years, 26% of patients in the combination arm and 14% of patients receiving monotherapy were still alive. Responses were seen regardless of platinum sensitivity or PD-L1 status. Based on these findings nivolumab with or without ipilimumab was recently added to the National Comprehensive Cancer Network® Guidelines for the treatment of extensive stage (ES)-SCLC. The combination of these two immunotherapies could be applied if there is a relapse of the disease <6 months.

At this year’s ASCO meeting, the presented data of the randomized Phase II cohort were 3‑month PFS and OS (PFS 18% for nivolumab and 30% for the combination arm). This is too early for interpretation but ORR of the randomized cohort was equal to the phase 1 cohort so maybe we will see promising results later this year.

## Maintenance therapy with pembrolizumab

In patients with ES-SCLC, median PFS after initial chemotherapy lies usually around 2 months. The phase II trial by Gadgeel et al. investigated if PFS could be extended by maintenance therapy with pembrolizumab. With only a median PFS of 1.4 months the study did not achieve the primary endpoint. However, exploratory analysis showed that patients with higher PD-L1 expression at the stromal interface had better outcomes. Further trials are needed to define the role of pembrolizumab in this setting.

The ECOG-ACRIN 2511 study evaluated the combination of the PARP-inhibitor (enzyme poly ADP ribose polymerase) veliparib with cisplatin/etoposide doublet as first-line treatment in ES-SCLC. Median PFS was 6.1 months for patients receiving veliparib and 5.5 months for patients receiving placebo (unstratified HR: 0.75; 1‑sided *p* = 0.06). Unfortunately, no biomarker analyses were planned so the question remains if analyzing gene signatures for DNA repair could define a subgroup of patients who benefit significantly.
